# Evaluation of the Functional and Nutritional Properties of Alpha-Amylase-Modified Cassava Starch in Breadmaking

**DOI:** 10.3390/foods15122197

**Published:** 2026-06-18

**Authors:** Vanessa Abad-Quevedo, Fabiola Cornejo, Pedro Maldonado-Alvarado

**Affiliations:** 1 Department of Food Science and Biotechnology, Escuela Politécnica Nacional, Quito 17-01-2759, Ecuador; 2 Facultad de Ingeniería en Mecánica y Ciencias de la Producción, Escuela Superior Politécnica del Litoral, ESPOL, Campus Gustavo Galindo, Km 30.5 Vía Perimetral, Guayaquil 090902, Ecuador; fcornejo@espol.edu.ec

**Keywords:** cassava, modified starch, alpha-amylase, gluten-free, breadmaking

## Abstract

Few strategies have been developed to mimic and control the supramolecular degradations induced by spontaneous fermentation in sour cassava starch, which are partly responsible for its characteristic expansion capacity in breadmaking, and their effectiveness has remained limited. In this context, the objective of this study was to evaluate the effect of adding α-amylase on the functional and nutritional properties of cassava starch used in breadmaking. Cassava starch from the INIAP 651 variety was modified with different α-amylase dosages (0, 2, 4, 6, 8, and 9 U/g α-amylase for 20 min), followed by hydration and pre-gelatinization before baking. Determinations of the specific volume of the bread (SV), dough characterization by Mixolab, pasting properties using a rheometer, and nutritional properties were performed. The treatment with 6 U/g α-amylase showed the best functional properties, achieving the highest SV (4.28 mL/g), C3 (1.67 Nm), C4 (1.11 Nm), and peak viscosity (6550 mPa·s), as well as the lowest setback (1526 mPa·s). In contrast, the treatment with 9 U/g α-amylase exhibited the most favorable nutritional profile, with the lowest estimated glycemic index (51.25) and rapidly digestible starch (15.85 g/100 g). These results confirm that controlled α-amylase dosing modulates cassava starch functionality for breadmaking and glycemic control.

## 1. Introduction

Starch is the primary storage polysaccharide in plants and a fundamental component in human nutrition and food systems. However, native starches often exhibit technological limitations, including low thermal stability, limited solubility, and poor expansion capacity, which restrict their application in bakery products. To overcome these constraints, a range of modification strategies—physical, chemical, enzymatic, and biological—has been explored. Among them, enzymatic modification has gained increasing attention for its precision and controllability, enabling targeted depolymerization and structural reorganization to tailor starch functionality [1,2].

Sour cassava starch is a modified starch obtained through spontaneous fermentation of cassava starch for approximately 30 days, followed by sun drying for about 12 h [3].

This process induces complex structural changes at both the molecular and supramolecular levels, including partial hydrolysis, acidification, and oxidative reactions, which are largely responsible for its distinctive capacity for expansion in gluten-free baking. Despite its technological advantages, the inherent variability of fermentation conditions leads to inconsistent functional properties, limiting reproducibility and broader industrial application [4,5].

Efforts to reproduce these properties have focused on both molecular and supramolecular modification strategies. At the molecular level, controlled acidification—particularly using lactic acid—combined with UV radiation has been shown to enhance expansion during baking [6]. Similarly, oxidative treatments with agents such as hydrogen peroxide and sodium hypochlorite promote depolymerization, the formation of carbonyl and carboxyl groups, a reduction in paste viscosity, and the inhibition of retrogradation, all of which contribute to improved expansion behavior [7,8].

At the supramolecular level, structural rearrangements within starch granules have been induced by treatments with organic acids (e.g., lactic, acetic, and propionic acids) followed by drying, either sun-drying or oven-drying. These modifications affect rheological behavior and vapor retention capacity, ultimately influencing bread specific volume [9,10]. Although these approaches can partially reproduce the functional properties of sour cassava starch, the combined effects of fermentation and sun drying have not yet been fully replicated, highlighting the complexity of the structural transformations involved [3,8].

Enzymatic approaches offer a more targeted mechanism for modifying starch structure. α-Amylase selectively hydrolyzes α-1,4 glycosidic bonds, promoting controlled depolymerization of starch chains and inducing structural rearrangements that affect granule integrity, water absorption, and pasting behavior. Recent studies have demonstrated that α-amylase treatment can significantly modify starch functionality by altering molecular weight distribution and enhancing water–starch interactions without completely disrupting the granular structure [1,11]. In addition, enzymatic hydrolysis has been associated with changes in starch digestibility, influencing the proportion of rapidly and slowly digestible fractions and, consequently, the glycemic response [12]. In addition to technological effects, enzymatic hydrolysis can influence starch digestibility by altering the proportions of rapidly and slowly digestible fractions, with direct implications for glycemic response.

Despite these advances, studies addressing the use of α-amylase to mimic fermentation-induced structural modifications in cassava starch remain limited, particularly when both technological performance and nutritional attributes are evaluated simultaneously in gluten-free bread systems. Maldonado-Alvarado et al., 2023 [13] demonstrated the potential of enzymatic hydrolysis at high α-amylase concentrations (100 U/g substrate); however, at these levels, a reduction in bread specific volume was observed, likely due to structural weakening from excessive depolymerization (2023).

The originality of the present study lies in the use of controlled enzymatic hydrolysis to mimic fermentation-induced structural changes in cassava starch, by evaluating the effects of moderate α-amylase levels and their simultaneous impact on technological and nutritional properties in a real gluten-free breadmaking system. Therefore, the aim of this study was to evaluate the effect of α-amylase treatment on the functional and nutritional properties of cassava starch, with particular emphasis on breadmaking performance and starch digestibility.

## 2. Materials and Methods

### 2.1. Raw Material

Cassava roots of the INIAP 651 variety (derived from clone CM 1335-4) were donated by the National Institute of Agricultural Research (INIAP), Portoviejo Experimental Station—Ecuador.

### 2.2. Starch Extraction and Processing

The starch was obtained from a cassava starch production plant or ‘Rallandería’ located in Manabí province from Ecuador. To obtain the cassava starch, the roots were first washed and peeled to remove soil residues and impurities. The cassava pulp was then grated to release the starch granules, followed by washing and filtering to obtain an aqueous suspension. This suspension was decanted in channels to separate the starch, which was subsequently sun-dried for 12 h to obtain non-fermented sun-dried cassava starch (NS).

### 2.3. Starch Modification

The selection of α-amylase dosages was based on preliminary experimental trials informed by previous findings by Maldonado-Alvarado et al. (2023) [13]. Initial assays focused on intermediate enzyme levels (10, 20, and 30 U/g α-amylase), which were directly evaluated in breadmaking through specific volume measurements. These trials revealed a consistent decrease in bread expansion with increasing enzyme concentration, confirming the system’s sensitivity to enzymatic dosage. Based on this response, a lower range of α-amylase concentrations (2, 4, 6, 8, and 9 U/g α-amylase) was selected to more accurately characterize the dose–response behavior under controlled hydrolysis conditions.

The procedure was adapted from a protocol reported previously [5]. First, the moisture content of the NS was adjusted to 80% (previously optimized value), starting from an initial value of 12%. For this purpose, each NS sample was divided into two fractions. The first represents 25.52%, and the second represents the remaining 74.48%. The first portion (25.52% NS) was partially gelatinized by adding 32.26% boiling water. Simultaneously, the remaining starch portion (74.48% NS) was mixed with 67.74% water at 37 °C and manually homogenized. Subsequently, a solution of α-amylase (Sigma-Aldrich, A3176, St. Louis, MO, USA) was prepared and maintained at 37 °C. This enzymatic solution was added to the mixture at concentrations of 0, 2, 4, 6, 8, and 9 U/g α-amylase. The reaction was allowed to proceed for 20 min at room temperature (previously optimized time). The final obtained product was used to determine specific volume and nutritional analyses.

In contrast, for analyses conducted directly on the modified starch, including dough characterization and pasting property measurements, an additional step was required to terminate the enzymatic reaction. For this purpose, 96% ethanol was added at a ratio of 1 mL per g of modified starch [14], followed by manual homogenization for 1 min to ensure uniform contact throughout the sample. The sample was then oven-dried (Rebelk, RS-40, Barcelona, Spain) at 40 °C for 48 h to remove residual moisture and promote ethanol evaporation. All experiments were conducted in triplicate using independent replicates.

### 2.4. Bread Preparation and Specific Volume Determination

Using the modified starch obtained in Section 2.3, three bread samples were prepared from dough portions of approximately 10 g each. These portions were placed on separate trays and baked in an oven (Unox Stefania XFT113, Cadoneghe, Italy) for 11 min at 260 °C. The baked doughs were cooled at room temperature for 24 h before determining specific volume. Bread volume was determined by the birdseed displacement method according to the AACC Approved Methods [15]. The bread weight was measured using a Radwag WTC 2000 scale (Radom, Poland) after 24 h of baking. Specific volume was calculated in mL/g by dividing the measured volume (mL) by the mass (g).

### 2.5. Dough Rheological Analysis

Using the MIXOLAB 2 equipment (Chopin Technologies, Villeneuve-la-Garenne, France), dough characteristics under kneading conditions with specific time and temperature profiles were determined. The Chopin+ protocol from the device software (Mixolab, software version 2, Chopin Technologies, Villeneuve-la-Garenne, France, 2012) was used to analyze the different treatments. The tests required approximately 50 g of sample (amount varying with dough moisture content) and were mixed at 80 rpm with the corresponding water content designated by the system. The parameters obtained were starch gelatinization (C3), gel stability under controlled temperature (C4), and starch retrogradation (C5). Additionally, C3−C4 were calculated due to them being considered indicators of the thermal and mechanical weakening of the starch paste after gelatinization, whereas C5−C4 were calculated because of their use to estimate the extent of starch reassociation during cooling [16]. Samples with a moisture content of approximately 5–8% were analyzed at 80% hydration (prior-optimized moisture). Cassava starch contains no gluten; therefore, data from C1 and C2 were excluded from this study.

### 2.6. RVA Pasting Analysis

Pasting analyses were performed using an RVA (Perten, Instruments, Springfield, IL, USA). The protocol was as follows: initial holding at 50 °C for 1 min, heating from 50 °C to 90 °C over 4 min, holding at 90 °C for 4 min, and cooling from 90 °C to 50 °C over 4 min, with continuous stirring at 160 rpm. From the pasting curves, the following parameters were obtained: peak viscosity, pasting temperature, holding strength, and final viscosity [17]. Two additional parameters were calculated: breakdown, the difference between peak viscosity and holding strength, and setback, the difference between final viscosity and holding strength.

### 2.7. Total Starch

Total starch content was determined in the final bread sample. Before the analyses for total starch, resistant starch, and starch digestibility, soluble sugars were removed. For sugar removal, 0.1 g of the sample was weighed, and 2 mL of 80% ethanol was added. The mixture was incubated in a water bath at 85 °C for 15 min with agitation at 150 rpm, then centrifuged at 3000× *g* rpm for 10 min. The resulting precipitate was placed in a drying oven (Rebelk, RS-40, Barcelona, Spain) until the ethanol was completely evaporated.

Total starch content was determined using the protocol provided by Megazyme et al. (2024) [18]. A 0.5 g sample was weighed, mixed with 10 mL of 80% *v*/*v* ethanol, incubated at 85 °C for 10 min at 400 rpm, and then centrifuged. The sample was then kept in a water bath with agitation at 85 °C for 5 min, followed by centrifugation at 1000× *g* for 10 min. Finally, the pellet was dried in a drying oven (Rebelk, RS-40 Barcelona, Spain) until ethanol was fully evaporated or the moisture content reached approximately 6%. Once dry, the remaining pellet was ground and sieved through a 70-mesh screen.

Total starch content was quantified using the Megazyme assay kit (AA/AMG, K-TSTA) (Megazyme International Ltd., Wicklow, Ireland), and absorbance was measured at 510 nm using a UV-Visible spectrophotometer (Thermo Scientific 201 Evolution, Madison, WI, USA). The total starch percentage was calculated based on the amount of glucose released during hydrolysis. A conversion factor is generally used to convert the measured glucose into a starch equivalent. A commonly used factor is that starch yields approximately 0.9 times as much glucose as the amount obtained, due to structural differences between starch and glucose.

### 2.8. Resistant Starch

Resistant starch content was determined in the final bread samples using the Resistant Starch Megazyme assay kit (Megazyme International Ltd., Wicklow, Ireland). Absorbance was measured at 510 nm using a UV–Visible spectrophotometer (Thermo Scientific 201 Evolution, Madison, WI, USA). Resistant starch was calculated as the difference between the amount of starch present before enzymatic digestion (total starch) and the amount of glucose released during enzymatic hydrolysis.

### 2.9. Estimated Glycemic Index (eGI)

The procedure followed was based on a previously reported method, with minor adjustments to suit the conditions of this study [19]. Bread samples were first ground (Hamilton Beach, Glen Allen, VA, USA) and sieved through a 70-mesh screen (212 µm) to obtain a uniform particle size. Starch hydrolysis was then evaluated in vitro using a modified version of the method described by Goñi et al. (1997) [20], employing a commercial enzymatic kit (Megazyme, Wiclow, Ireland). Digestion was carried out under controlled temperature and pH conditions using pancreatic α-amylase and amyloglucosidase, allowing consistent monitoring of hydrolysis kinetics. Absorbance readings were taken at 510 nm using a microplate reader (Biotek Instruments, Winooski, VT, USA).

Hydrolysis kinetics were described using a first-order model:

(1) $$C\;=\;C_{\infty }\;(1\;-\;e^{-kt})$$

where *C* represents the percentage of starch hydrolyzed at time *t*, *C*_∞_ corresponds to the equilibrium hydrolysis reached after 150 min, and *k* is the kinetic constant.

The hydrolysis index (HI) was calculated as the ratio of the area under the hydrolysis curve (AUC) for each sample to that of white bread, used as the reference. The AUC was determined considering the fractions of rapidly digestible starch (0–20 min) and slowly digestible starch (20–120 min), following the criteria commonly used in starch digestibility studies [20].

The estimated glycemic index (eGI) was calculated from HI using the empirical equation:

(2) eGI=8.198+0.862 HI

It is important to emphasize that eGI values are indirect estimates and should be interpreted as approximations of the in vivo glycemic response. This approach allows a consistent comparison of relative differences in starch digestibility under controlled experimental conditions.

### 2.10. Statistical Analysis

Statistically significant differences between the mean values of three repetitions were determined using analysis of variance (ANOVA), followed by Fisher’s test with a 95% confidence level. Statistical analyses were performed using STATGRAPHICS CENTURION XIX software 19.1.03.

## 3. Results and Discussion

### 3.1. Breadmaking Ability

The specific volume (SV) of bread is a main indicator of its expansion capacity, reflecting the starch matrix’s ability to retain gas during baking. The SV of bread was within the range commonly reported for cassava starches [3], although slightly lower than the values described for commercial sour cassava starch and some Latin American varieties [21,22]. The variation observed in the results may be attributed to intrinsic and environmental factors, including genotype, cultivation altitude, climatic conditions, and possibly amylose content, all of which exert a decisive influence on the expansion capacity of sour cassava starch.

The highest specific volume was obtained at 6 U/g α-amylase. This behavior is associated with partial hydrolysis of starch chains, which enhances water absorption and promotes granule swelling. Under these conditions, a viscoelastic network forms, which retains gas during baking, thereby contributing to greater product expansion [1,5,11].

The specific volume results obtained for the different α-amylase treatments are presented in Table 1.

Treatments with 2 and 9 U/g α-amylase did not differ significantly from the control. At a low-enzyme dosage of 2 U/g α-amylase, the enzymatic activity is insufficient to induce structural modifications relevant to gas retention [22]. In contrast, at 9 U/g of α-amylase, excessive hydrolysis leads to the formation of low-molecular-weight compounds that reduce system viscosity and weaken the dough structure, thereby negatively affecting bread expansion [23].

### 3.2. Dough Characterization

The Mixolab parameters provided insight into the effect of α-amylase hydrolysis on cassava starch behavior under thermo-mechanical conditions, particularly during gelatinization, paste stability, and cooling-induced reassociation [24,25,26].

The highest C3 value observed at 6 U/g α-amylase suggests that moderate enzymatic hydrolysis favored water accessibility and granule swelling without severely disrupting starch structure. This condition promoted the development of viscosity during heating, indicating that the starch matrix retained enough structural continuity to sustain viscosity development [27]. Similar effects have been associated with controlled structural modification of starch, where partial depolymerization increases water–polymer interactions while maintaining granular functionality [28]. In contrast, lower enzyme levels appeared insufficient to produce relevant structural changes, whereas higher dosages probably caused excessive chain fragmentation, reducing granule integrity and limiting viscosity development [2,29,30].

A similar trend was observed for C4, which reflects the stability of the gelatinized paste under combined thermal and mechanical stress. The highest C4 value at 6 U/g α-amylase indicates improved resistance to breakdown, suggesting that this treatment generated a starch matrix capable of maintaining structural integrity during heating. This behavior is consistent with a balanced degree of hydrolysis, where increased molecular mobility does not compromise chain interactions. Conversely, the reduction in C4 at higher enzyme levels (8 and 9 U/g U/g α-amylase) suggests decreased paste stability, likely due to extensive depolymerization and the formation of low-molecular-weight fragments that weaken network cohesion [2,31].

The Mixolab parameters obtained for the different treatments are summarized in Table 2.

The C3–C4 difference provides additional insight into the susceptibility of the gelatinized starch to structural disintegration under sustained heating and shear. Larger differences observed at higher enzyme dosages indicate greater loss of consistency, consistent with increased fragmentation of the starch matrix. In contrast, the smaller C3–C4 difference at 6 U/g α-amylase suggests limited breakdown after gelatinization, supporting the formation of a more stable viscoelastic system. This balance between swelling and stability is particularly relevant in breadmaking, as it contributes to gas retention and expansion during baking [2,29,30].

During cooling, C5 and C5–C4 reflected the ability of starch chains to reassociate and form ordered structures. The higher C5 response at 6 U/g α-amylase indicates that controlled hydrolysis generated chain segments with sufficient mobility to reorganize during cooling, favoring retrogradation. This interpretation agrees with previous reports showing that retrogradation depends on the availability, length, and mobility of starch chains after gelatinization [27,28]. In contrast, lower enzyme dosages likely caused minimal structural modification, whereas excessive hydrolysis at 9 U/g α-amylase produced shorter fragments with reduced capacity for effective reassociation [24].

Overall, the Mixolab results indicate that cassava starch behavior was strongly dependent on the degree of enzymatic hydrolysis. Treatment with 6 U/g α-amylase provided the most favorable balance among gelatinization, paste stability, and molecular reassociation, whereas lower dosages caused limited modification, and higher dosages led to excessive structural weakening. These findings support the idea that controlled α-amylase treatment can modulate the thermo-mechanical behavior of cassava starch and help explain the improved bread expansion observed at the same enzyme level.

### 3.3. Pasting Properties

The pasting profile evaluation allowed assessment of the effect of α-amylase on the gelatinization behavior, thermal stability, and retrogradation of native starch. The pasting properties obtained for the evaluated treatments are presented in Table 3.

Pasting temperature remained relatively constant across treatments, indicating that enzymatic modification did not substantially affect the crystalline regions of amylopectin responsible for the onset of gelatinization. This suggests that α-amylase activity was mainly directed toward amorphous domains, which are more susceptible to hydrolysis and primarily influence viscosity-related parameters rather than thermal transition points [3,32].

Peak viscosity (PV) values showed a clear dependence on α-amylase dosage, reflecting changes in the swelling capacity of starch granules during heating. The highest PV values observed at intermediate enzyme levels (4–6 U/g α-amylase) indicate that controlled hydrolysis enhanced water accessibility and promoted granule swelling without compromising their structural integrity.

The reduction in PV at higher enzyme dosages (8–9 U/g α-amylase) suggests that extensive hydrolysis limited the swelling capacity of starch granules. This effect is likely associated with the formation of shorter chains and increased solubilization of starch components, which reduce the ability of the granules to absorb water and expand during heating [11].

A similar pattern was observed for holding strength (HS), which reflects the stability of the gelatinized system under thermal and mechanical stress. The increased HS at 6 U/g α-amylase indicates improved hot-paste stability, likely due to a balanced modification of the starch structure that maintains sufficient intermolecular interactions. In contrast, the reduced stability at higher enzyme levels can be attributed to diminished structural cohesion resulting from more extensive chain degradation [3,32].

The relationship between PV, HS, and breakdown (BD) further illustrates the impact of enzymatic treatment on granule integrity. Higher BD values at intermediate enzyme concentrations indicate that granules underwent substantial swelling, followed by increased susceptibility to shear-induced disintegration. Conversely, the lower BD values observed at higher enzyme dosages suggest that prior structural weakening limited swelling, reducing the extent of subsequent breakdown during pasting. This shift reflects a transition from swelling-driven behavior to one dominated by pre-existing structural degradation [3,33].

The setback (SB) values provided information on the ability of starch chains to reorganize into ordered structures. Moderate enzyme levels (2–4 U/g α-amylase) promoted retrogradation, suggesting that hydrolysis generated chain fragments with sufficient length and mobility to reassociate effectively. At 6 U/g α-amylase, the reduction in SB indicates that further depolymerization began to limit this process, while at higher enzyme levels, the persistence of low SB values reflects restricted molecular reorganization due to the predominance of shorter chains [6,33,34,35].

Overall, under conditions of excess hydration, the extent of enzymatic hydrolysis governed the balance among granule swelling, viscosity development, and structural stability. Intermediate α-amylase levels promoted enhanced swelling and paste stability, whereas higher dosages led to extensive structural degradation, limiting both viscosity and retrogradation. These results complement the thermo-mechanical behavior observed in the Mixolab analysis and help explain the functional performance of cassava starch in breadmaking systems.

### 3.4. Nutritional Properties

The nutritional profile of cassava starch was evaluated through its digestible fractions and estimated glycemic index (eGI), providing insight into the relationship between enzymatic modification and starch digestibility. It is important to note that the eGI values obtained in this study are based on in vitro hydrolysis and should be interpreted as approximations of in vivo glycemic response rather than direct physiological measurements.

Enzymatic treatment altered starch digestibility primarily as a function of hydrolysis intensity. Limited modification at 2–4 U/g α-amylase did not significantly affect the distribution of starch fractions, indicating that structural changes at these levels were insufficient to modify enzyme accessibility during digestion.

Higher enzyme dosages (8–9 U/g) promoted structural rearrangements that reduced the proportion of non-resistant starch (NRS), suggesting a lower fraction of rapidly hydrolyzed carbohydrates. This behavior is consistent with alterations in starch organization that can restrict enzymatic attack despite partial depolymerization [36,37].

The nutritional properties and estimated glycemic index values are presented in Table 4.

Changes in total starch (TS) further support the occurrence of enzymatic degradation. The reduction observed at higher α-amylase levels can be attributed to the formation of soluble dextrins and oligosaccharides that are not fully quantified as starch by conventional enzymatic assays. This phenomenon has been widely reported in enzymatically modified starch systems and reflects the conversion of polymeric structures into low-molecular-weight fractions [38,39].

The distribution of digestible fractions revealed a progressive shift in starch organization. Resistant starch (RS) decreased as enzyme dosage increased, indicating that hydrolysis disrupted the ordered and semi-crystalline regions that normally resist enzymatic digestion. As these structures were degraded, starch became more susceptible to hydrolysis, reducing the fraction that escapes digestion. From a nutritional perspective, this reduction may limit the physiological benefits associated with RS, such as attenuation of postprandial glucose response [40,41].

Rapidly digestible starch (RDS) showed only minor variations across treatments, with a noticeable reduction only at the highest enzyme level. This suggests that extensive hydrolysis may alter starch organization, reducing immediate enzymatic accessibility, potentially due to the formation of rearranged or less accessible structures during processing. Meanwhile, slowly digestible starch (SDS) remained largely unaffected, indicating that the structural features governing intermediate digestion rates were relatively stable under the conditions evaluated [40,42,43].

These changes in starch fractions were reflected in the estimated glycemic index (eGI). Based on the estimated values, all samples fell within the low-to-moderate glycemic index range according to FAO/WHO classification criteria [44]; however, a decrease in eGI was observed at higher enzyme dosages. This trend suggests that structural modifications induced by α-amylase treatment influenced hydrolysis kinetics, limiting the rate of glucose release under in vitro conditions [45,46].

The relationship between enzymatic modification and estimated glycemic response highlights a functional trade-off within the system. While higher enzyme levels reduced eGI and modified digestibility patterns, they were also associated with structural degradation that may compromise technological performance. Conversely, intermediate treatments preserved functional properties but showed limited impact on digestibility. This balance underscores the importance of controlling enzymatic hydrolysis to tailor both nutritional and technological attributes in cassava-based products.

Cyanogenic glycoside content was not determined in the present study. The raw material corresponded to the INIAP 651 variety, for which an initial hydrocyanic acid (HCN) content of 0.230 mg·g^−1^ in fresh roots has been reported by Castro-Moreira et al. (2021) [47]. This value decreases to 0.040 mg·g^−1^ after processing steps—including peeling, grating, washing, sedimentation, and drying—representing an overall reduction of approximately 85–88%.

## 4. Conclusions

This study indicates that controlled enzymatic modification of cassava starch with α-amylase is an effective approach to modulate both functional and nutritional properties relevant to gluten-free breadmaking.

From a technological perspective, treatment with 6 U/g α-amylase provided optimal performance, as evidenced by improved bread expansion and favorable thermo-mechanical behavior, indicating a suitable balance between starch depolymerization and structural integrity.

The highest enzyme levels evaluated in this work (9 U/g α-amylase) led to a modified digestibility profile, characterized by reduced rapidly digestible starch and lower estimated glycemic index (eGI) values, reflecting slower starch hydrolysis under in vitro conditions.

The range of 6–9 U/g α-amylase defines a practical window in which starch functionality and digestibility can be modulated according to the intended application.

These findings highlight the potential of controlled α-amylase hydrolysis as a strategy to tailor cassava starch performance, supporting its use as an ingredient for the development of gluten-free bakery products with improved technological properties and adjusted nutritional characteristics.

## Figures and Tables

**Table 1:** Bread Specific volume.

NS + U/g α Amylase	Specific Volume (mL/g)
0	1.93 ± 0.04 ^a^
2	1.68 ± 0.15 ^a^
4	2.90 ± 0.32 ^b^
6	4.28 ± 0.28 ^c^
8	2.64 ± 0.35 ^b^
9	1.88 ± 0.02 ^a^

*n* = 3 Values followed by different letters (a–c) in the same row denote statistically significant differences (LSD, *p* < 0.05). NS = non-fermented sun-dried cassava starch.

**Table 2:** Dough characterization.

NS + U/g α Amylase	C3 (Nm)	C4 (Nm)	C3–C4 (Nm)	C5 (Nm)	C5–C4 (Nm)
0	1.54 ± 0.06 ^d^	0.95 ± 0.03 ^d^	0.59 ± 0.00 ^b^	1.89 ± 0.02 ^c^	0.94 ± 0.01 ^d^
2	1.37 ± 0.02 ^a^	0.78 ± 0.00 ^b^	0.59 ± 0.02 ^ab^	1.28 ± 0.00 ^a^	0.49 ± 0.01 ^a^
4	1.45 ± 0.00 ^c^	0.83 ± 0.01 ^c^	0.61 ± 0.00 ^b^	1.36 ± 0.00 ^ab^	0.52 ± 0.00 ^ab^
6	1.67 ± 0.00 ^e^	1.11 ± 0.02 ^e^	0.55 ± 0.01 ^a^	2.19 ± 0.12 ^d^	1.07 ± 0.11 ^e^
8	1.45 ± 0.02 ^c^	0.73 ± 0.01 ^a^	0.72 ± 0.01 ^c^	1.43 ± 0.05 ^b^	0.69 ± 0.05 ^c^
9	1.41 ± 0.00 ^b^	0.71 ± 0.00 ^a^	0.69 ± 0.00 ^c^	1.30 ± 0.00 ^a^	0.59 ± 0.00 ^b^

*n* = 3 Values followed by different letters (a–e) in the same row denote statistically significant differences (LSD, *p* < 0.05). NS = non-fermented sun-dried cassava starch.

**Table 3:** Pasting Properties.

NS + U/g α Amylase	Pasting Temperature (°C)	Peak Viscosity (mPa·s)	Trough (mPa·s)	Breakdown (mPa·s)	Viscosity Final (mPa·s)	Set Back (mPa·s)
0	70.10 ± 0.00 ^a^	4349 ± 32 ^a^	2414 ± 13 ^b^	1935 ± 19 ^a^	3173 ± 16 ^a^	758 ± 2 ^a^
2	71.27 ± 0.37 ^c^	5847 ± 40 ^c^	2619 ± 33 ^c^	3228 ± 74 ^c^	4206 ± 9 ^c^	1587 ± 24 ^c^
4	70.57 ± 0.32 ^bc^	6407 ± 213 ^d^	2868 ± 94 ^d^	3539 ± 119 ^d^	4522 ± 110 ^d^	1654 ± 16 ^d^
6	70.47 ± 0.37 ^ad^	6550 ± 239 ^d^	3025 ± 107 ^e^	3525 ± 132 ^d^	4551 ± 124 ^d^	1526 ± 17 ^b^
8	70.90 ± 0.00 ^c^	5868 ± 122 ^c^	2524 ± 120 ^bc^	3344 ± 2 ^c^	4117 ± 74 ^c^	1593 ± 46 ^c^
9	70.10 ± 0.00 ^a^	5328 ± 99 ^b^	2255 ± 24 ^a^	3003 ± 53 ^b^	3828 ± 77 ^b^	1503 ± 31 ^b^

*n* = 3 Values followed by different letters (a–e) in the same row denote statistically significant differences (LSD, *p* < 0.05). NS = non-fermented sun-dried cassava starch.

**Table 4:** Nutritional Properties.

NS + U/g α Amylase	NRS (g/100 g)	TS (g/100 g)	RS (g/100 g)	RDS (g/100 g)	SDS (g/100 g)	Egi
0	45.14 ± 2.98 ^c^	45.72 ± 2.97 ^c^	0.58 ± 0.01 ^d^	23.37 ± 0.30 ^b^	21.76 ± 0.30 ^abc^	58.98 ± 0.87 ^cd^
2	45.09 ± 1.84 ^c^	45.58 ± 1.83 ^c^	0.48 ± 0.01 ^c^	27.52 ± 0.59 ^b^	27.52 ± 0.59 ^c^	60.99 ± 0.66 ^d^
4	42.96 ± 0.55 ^bc^	43.44 ± 0.54 ^bc^	0.47 ± 0.01 ^bc^	27.52 ± 0.59 ^b^	15.44 ± 0.59 ^a^	60.11 ± 1.22 ^d^
6	42.12 ± 0.89 ^b^	42.57 ± 0.88 ^b^	0.44 ± 0.05 ^b^	26.78 ± 2.69 ^b^	15.33 ± 2.69 ^a^	57.48 ± 0.66 ^c^
8	40.86 ± 0.63 ^ab^	41.26 ± 0.64 ^ab^	0.39 ± 0.02 ^a^	22.98 ± 11.32 ^b^	17.87 ± 11.32 ^ab^	53.77 ± 3.29 ^b^
9	39.63 ± 0.95 ^a^	40.00 ± 0.94 ^a^	0.37 ± 0.00 ^a^	15.85 ± 0.98 ^a^	23.77 ± 0.98 ^bc^	51.25 ± 1.45 ^a^

*n* = 3 Values followed by different letters (a–d) in the same row denote statistically significant differences (LSD, *p* < 0.05).

## Data Availability

The original contributions presented in this study are included in the article. Further inquiries can be directed to the corresponding author.

## Abbreviations

The following abbreviations are used in this manuscript:

BD	Breakdown
C3	Gelatinization torque
C4	Stability during heating
C5	Retrogradation torque
eGI	Estimated glycemic index
FV	Final Viscosity
GI	Glycemic Index
HS	Holding Strength
LSD	Least significant difference
NRS	Non-Resistant Starch
NS	non-fermented sun-dried cassava starch
PV	Peak Viscosity
RVA	Rapid Visco Analyzer
RDS	Rapidly Digestible Starch
RS	Resistant Starch
SB	Setback
SDS	Slowly Digestible Starch
SV	Specific Volume
TS	Total Starch
